# The role of advanced glycation end products (AGEs) and the receptor for AGEs (RAGE) in hypertrophic obstructive cardiomyopathy

**DOI:** 10.1371/journal.pone.0328032

**Published:** 2025-07-24

**Authors:** Shengxu Li, Xuanye Bi, Quanxu An, Yuhang Li, Yazhe Tang

**Affiliations:** 1 Department of Plastic Surgery, The Fifth Clinical Medical College of Henan University of Chinese Medicine (Zhengzhou People’s Hospital), Zhengzhou, China; 2 Department of Cardiovascular Disease, State Key Laboratory of Cardiovascular Disease, Fuwai Hospital, National Center for Cardiovascular Diseases, Chinese Academy of Medical Sciences and Peking Union Medical College, Beijing, China; 3 Department of Cardiology, Henan Province Key Laboratory of Cardiac Injury and Repair, The First Affiliated Hospital of Zhengzhou University, Zhengzhou, China; Jordan University of Science and Technology Faculty of Medicine, JORDAN

## Abstract

**Background:**

Advanced glycation end products(AGEs)/RAGE(receptor for AGEs) play divergent roles in cardiovascular disease. Our study is to evaluate the correlation between AGEs/RAGE in circulation (soluble AGEs/RAGE, sAGEs/sRAGE) and in myocardium (mAGEs/mRAGE), and to explore their relationship with cardiac function and prognostic value in hypertrophic obstructive cardiomyopathy (HOCM).

**Methods:**

78 HOCM patients under septal myectomy were recruited. The soluble and myocardial AGEs/RAGE levels were determined by a commercial available ELISA kit at the time of baseline examination. Strain analysis in HOCM patients derived from cardiac magnetic resonance feature tracking, including global/septal radial strains (GRS/SRS), circumferential strains(GCS/SCS), and longitudinal strains(GLS/SLS). Histological fibrosis was assessed through masson’s staining as collagen volume fraction (CVF). All patients were followed up for a composite endpoint for a median duration of 3.8 years.

**Results:**

sAGEs/sRAGE were higher in HOCM patients than healthy controls(p = 0.025; p = 0.028). Log sRAGE was correlated with log mRAGE(r = 0.739, p < 0.01), CVF(r = −0.411, p < 0.01), GRS (r = 0.412, p < 0.01), GCS(r = 0.463, p < 0.01). Log mRAGE also showed a correlation with CVF(r = −0.439, p = 0.003), SRS (r = 0.4, p = 0.013) and SCS (r = 0.362, p = 0.03). Log mAGEs was correlated with log mRAGE(r = 0.376, p = 0.012). Multivariate COX analysis revealed that log sRAGE was a significant predictor for the occurrence of adverse events in HOCM patients(HR, 0.013; 95% CI, 0.001–0.313; p = 0.007).

**Conclusions:**

Circulating RAGE appears to act as a protective biomarker, as it is associated with better prognosis after septal myectomy, reducing fibrosis and improving cardiac function in HOCM patients. It is plausible that higher circulating RAGE levels may be derived from higher expression levels in the myocardium.

## 1. Introduction

Hypertrophic cardiomyopathy (HCM) is a clinically and genetically heterogeneous disorder. With the contemporary treatments for HCM, mortality due to the disease is low, and HCM patients have normal or nearnormal life expectancy without significant adverse events [1]. Hypertrophic obstructive cardiomyopathy (HOCM) is a clinical phenotype of HCM that presents with elevated left ventricular(LV) outflow gradient. Nonobstructive HCM are believed to experience a relatively stable clinical course without significant symptoms and high-risk profile [2]. However, patients with HOCM often experience significant symptoms such as exercise intolerance, chest pain, dyspnea, and syncope, and have a high risk of sudden death and heart failure, which can substantially deteriorate their health-related quality of life [3]. The pathophysiology of HOCM involves large amounts of functional proteins in response to stress or damage. However, the full understanding of the procession remains incomplete.

**Fig 1:**
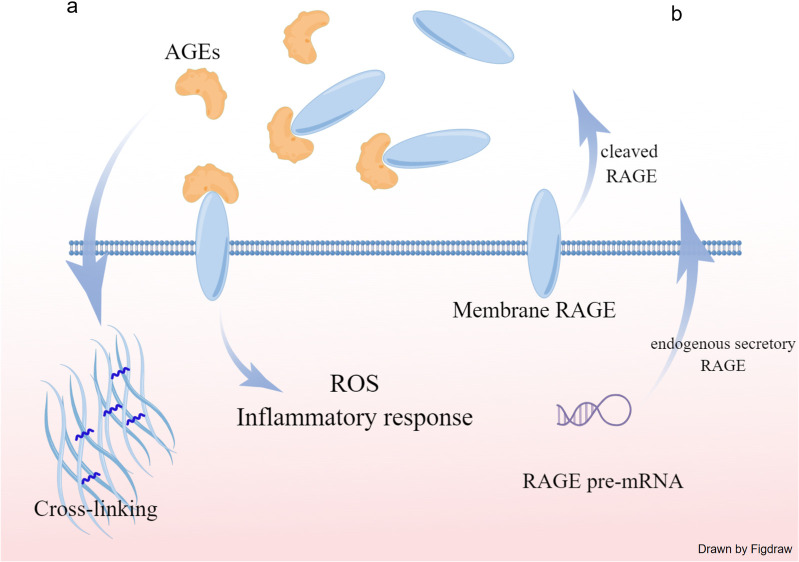
The functions of advanced glycation end products (AGEs) and the receptor for AGE (RAGE). (a)AGEs can lead to abnormal cross-linking of extracellular and intracellular proteins disrupting their normal structure and function. Furthermore, activation of AGE receptors can induce complex signaling pathways leading to increased inflammation, oxidative stress, and enhanced calcium deposition. (b) These two forms are the main contributors to sRAGE levels: endogenous secretory RAGE (esRAGE) formed by a splicing variant of RAGE gene, and cleaved RAGE (cRAGE), which comes from the degradation of membrane RAGE. sRAGE may contribute to the removal and neutralization of circulating ligands, thus functioning as a decoy.

Advanced glycation end products (AGEs) are a heterogeneous class of molecules. The synthesis of AGEs was mediated by non-enzymatic cross-linking between glucose and amino groups in proteins, lipids and nucleic acids [4]. Normally, AGEs form at a slow rate and tend to accumulate in the body during the aging process. However, under conditions of inflammation and oxidative stress, this process is accelerated, leading to increased levels of AGEs [5]. Elevated AGEs levels can lead to abnormal cross-linking of extracellular and intracellular proteins disrupting their normal structure and function. Furthermore, activation of AGE receptors can induce complex signaling pathways leading to increased inflammation, oxidative stress, and enhanced calcium deposition(Fig 1a) [6].

The receptor for AGEs (RAGE) is one of the most studied receptors for AGEs [7]. The transmembrane form of RAGE functions as a pattern recognition receptor, participating in cellular autophagy, apoptosis and inflammation [8]. Soluble RAGE(sRAGE) also can be found in human circulation. Opinions regarding the role of sRAGE are not always consistent. Previous studies have shown that elevated levels of sRAGE could have a protective anti-inflammatory effect in patients with coronary artery disease, Alzheimer’s disease, and hypertension [8–11]. These studies hypothesized that by competing with cell-surface RAGE for ligand binding, sRAGE may contribute to the removal and neutralization of circulating ligands, thus functioning as a decoy (Fig 1b) [12]. However, there is also some evidence indicating total sRAGE levels could be increased, rather than decreased, in some disease states such as end-stage renal disease and pulmonary arterial hypertension [13,14]. Therefore it is not clear the role of AGEs/RAGE in the progression of diseases.

Several studies have demonstrated potential associations between AGEs/RAGE and conditions such as heart failure, left ventricular hypertrophy, and cardiac fibrosis [15,16]. However, to date, there has been no systematic investigation of AGEs/RAGE in a single study. The current study aims to illustrate the role of AGEs/RAGE in HOCM patients by examining three key areas: 1) the correlation of AGEs/RAGE between circulation(soluble AGEs/RAGE, sAGEs/sRAGE) and myocardium(mAGEs/mRAGE), 2) if AGEs/RAGE are associated with cardiac function, and 3) if AGEs/RAGE have prognostic value for adverse events.

## 2. Methods

### 2.1. Study population

**Fig 2:**
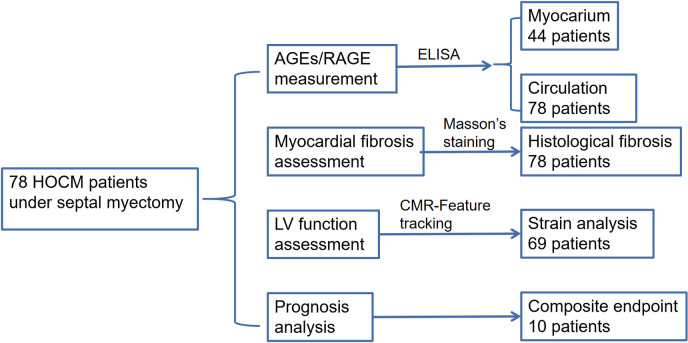
Flow chart of subject selection and analysis.

From 01/02/ 2019–30/09/2019, a total of 78 patients with HOCM results were enrolled at Fuwai Hospital, Chinese Academy of Medical Sciences (Fig 2). The diagnosis of HCM and assessment of preoperative indications are as previously described [14]. Exclusion criteria included cardiac valve diseases, history of acute myocardial infarction, liver/kidney dysfunction, or other severe chronic diseases.

Blood samples were collected from 35 healthy volunteers (14 males and 21 females; 50.8 ± 15.3 years) who were age- and sex-matched with the study cohort and who showed no evidence of cardiovascular-related diseases. These samples served as a control reference for the study. The study conforms to the principles of the Declaration of Helsinki. Written informed consent was obtained from all participants, with legally authorized representatives providing supplementary signatures for minors under 18 years of age following strict ethical review board protocols. The ethics committee of the Fuwai Hospital approved this research(Ethical Approval Number: 2015-ZX41).

### 2.2. Measurement of biochemical biomarkers

Serum AGEs/RAGE, myocardial AGEs/RAGE and NT-proBNP were assessed by enzyme-linked immunosorbent assay (ELISA). Additional details can be found in Supplemental material.

### 2.3. Quantification of myocardial fibrosis

Histological myocardial fibrosis was assessed by Masson’s trichrome staining on each myocardium slices. The collagen volume fraction (CVF) was calculated as the ratio of collagen-specific staining to the total area of the myocardium in each specimen.

### 2.4. CMR protocol and Analysis

All patients underwent MRI examination at 1.5 T (Magnetom Avanto, Siemens Healthineers) with an eight-channel cardiac coil before septal myectomy. LV mass and LV end-diastole and end-systole volume were measured using Argus software (version VA60C, Siemens Healthineers). Feature-tracking analysis was performed using the QStrain package (Medis Medical Imaging Systems). The global longitudinal strains(GLS) were obtained by tracking the long horizontal axis cines whereas the circumferential(GCS) and radial strains(GRS) were derived from the short-axis cines on the standard CMR steady-state free precession sequence. The septal radial(SRS), circumferential(SCS), and longitudinal strains(SLS) were defined as the mean of peak strain from the basal-anteroseptal and mid-anteroseptal segments.

### 2.5. Cardiac surgery

We applied extended septal myectomy evolving from the classic Morrow procedure. Additional details can be found in Supplemental material.

### 2.6. Follow-up and outcomes

All patients were followed up annually until April 2023 through phone conversation with patients or family members. A composite cardiovascular endpoint was defined as the primary outcome, which consisted of new-onset atrial fibrillation, ventricular tachycardia, hospitalization for heart failure, stroke, acute coronary syndrome and cardiovascular death.

### 2.7. Statistical analysis

Data are presented as the mean±SD for normally distributed continuous variables and as medians and interquartile ranges for non-normally distributed continuous variables. Differences between means were evaluated using paired and unpaired (for independent group comparisons) Student’s t tests for normally distributed data and the Mann–Whitney or Wilcoxon signed rank test for nonparametric data. The χ2 test was employed to compare categories of data. Logarithmic transformation of parameters was applied for linearity, as appropriate. Pearson correlation coefficients were used to investigate the relationships between the changes in parameters. Te composite endpoint was plotted according to the Kaplan–Meier method, and was compared using the log-rank test. Univariate and then multivariate analyses to determine the relative contribution of variables to overall mortality were examined by the Cox regression model. Statistical significance was defined as p < 0.05. All statistical analyses were performed using SPSS version 22 for Windows (SPSS, Inc., Chicago, Illinois).

## 3. Results

A total of 78 patients were recruited for the study. The patients were subsequently divided into two groups based on the median of serum RAGE value (high vs low sRAGE). The baseline clinical characteristics of the patients are described in Table 1. HOCM patients showed a similar age and gender composition between subgroups with high and low sRAGE. Patients with high sRAGE had a lower proportion of diabetes patients (12.5% vs. 87.5%, p = 0.028), as well as higher mRAGE(84.4(47–112.1)pg/ml vs 30.1(16.9–43.7)pg/ml, p < 0.001) and lower CVF values (10 ± 3.8% vs. 13 ± 5.4%, p = 0.007), compared to patients with low sRAGE.

**Table 1:** Demographic and clinical characteristics of the HOCM patients.

	Overall patients (n = 78)	Low sRGAE (n = 39)	High sRGAE (n = 39)	p value
Age (year)	50.7 ± 12.9	51.5 ± 12.2	49.8 ± 13.7	0.572
Age at diagnosis(year)	35.9 ± 17	37.1 ± 16.7	34.7 ± 17.3	0.543
Male (%)	47.4	56.8	43.2	0.257
Body mass index(Kg/m2)	24.2 ± 3.1	24.5 ± 2.7	23.9 ± 3.5	0.42
Atrial fibrillation (%)	12.8	60	40	0.368
NSVT (%)	15.4	50	50	1
NYHA class III/IV (%)	29.5	34.8	65.2	0.082
Peak LVOT gradient, mmHg	77.5(64-104.3)	74(64-100)	86(71-112)	0.228
Family history HCM of SCD (%)	9	42.9	57.1	0.5
Hypertension (%)	30.8	45.8	54.2	0.624
Diabetes (%)	10.3	87.5	12.5	0.028
Beta blockers (%)	91	50.7	49.3	0.5
ACEI/ARB (%)	5.1	50	50	0.692
Nondihydropyridine CCB (%)	6.4	80	20	0.179
CVF (%)	11.5 ± 4.9	13 ± 5.4	10 ± 3.8	0.007
NT-proBNP (pg/ml)	836.7(487.4-21666.3)	1213(534.5-2212)	733.3(400-1582)	0.199
sRAGE (pg/ml)	1069.9(751.3-1685.4)	752(580-944.3)	1660.4(1335.9-2198.2)	–
mRAGE(pg/ml)	43.8(20.8-83.6)	30.1(16.9-43.7)	84.4(47-112.1)	<0.001
sAGE (μg/ml)	128(84.4-182.7)	125.5(84.1-185.4)	144.1(84.4-181.8)	0.545
mAGE (μg/ml)	0.78(0.42-1.08)	0.65(0.38-1.06)	0.85(0.5-1.1)	0.353

ACEI, angiotensin-converting enzyme inhibitor; ARB angiotensin receptor blocker; AGEs, advanced glycation end products; CCB, calcium channel blockers; CMR, cardiac MRI; CVF, collagen volume fraction; HCM, hypertrophic cardiomyopathy; HOCM, hypertrophic obstructive cardiomyopathy; LV, left ventricular; LVOT, left ventricular outflow tract; NYHA, New York Heart Association; NSVT, nonsustained ventricular tachycardia; NT-proBNP, N-terminal pro-brain natriuretic peptide; RAGE, receptor for AGEs; SCD,sudden cardiac death.

### 3.1. AGEs/RAGE in HOCM patients

**Fig 3:**
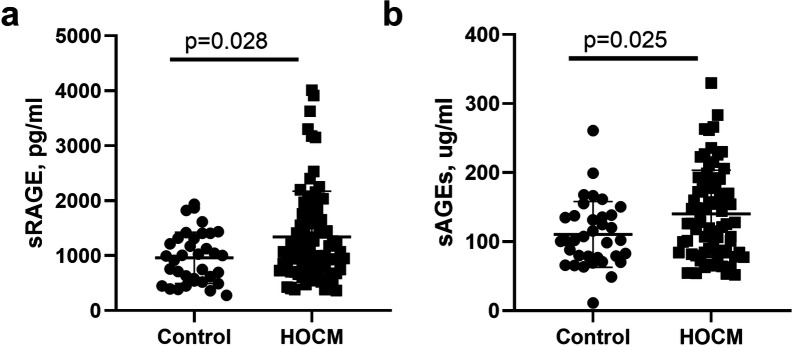
Comparisions of sRAGE(a) and sAGEs(b) between healthy controls and HOCM patients.

HOCM patients showed slightly higher sRAGE[1069.9(751.3–1685.4)pg/ml vs 993.3(537.7–1319.8)pg/ml, p = 0.028; Fig 3a] and sAGEs[128(84.4–182.7)ug/ml vs 102(76.7–137.6)ug/ml, p = 0.025; Fig 3b] than healthy controls.

**Fig 4:**
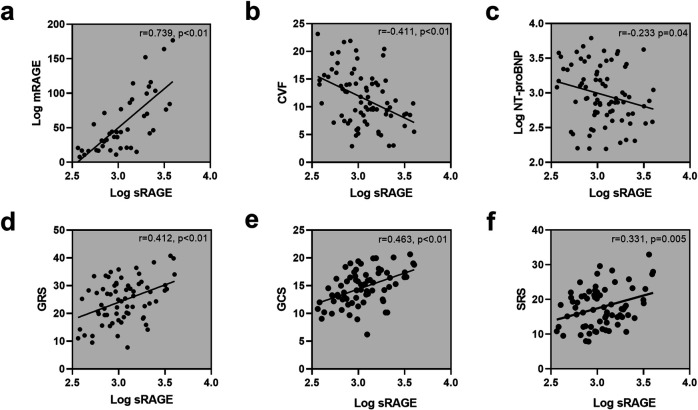
Correlations of log sRAGE with biochemical variables and LV function in HOCM patients.

Log sRAGE was positively correlated with log mRAGE(r = 0.739, p < 0.01; Fig 4a), but negatively correlated with log NT-proBNP(r = −0.233 p = 0.04; Fig 4c) and CVF(r = −0.411, p < 0.01; Fig 4b). Log mRAGE also showed an inverse correlation with CVF(r = −0.439, p = 0.003, Fig 4g). Log mAGEs was only correlated with log mRAGE(r = 0.376, p = 0.012, Fig 4h). However, log sAGEs did not show any statistically significant correlation with circulating or myocardial variables.

### 3.2. Associations between CMR parameters and AGEs/RAGE

Compared with HOCM patients with low sRAGE, patients with high sRAGE showed higher GRS(26.3 ± 7.5% vs 23 ± 7.7%, p = 0.076) and GCS(15.5 ± 3.2% vs 13.8 ± 3%, p = 0.076; Table 2). While no difference was found in LV structure parameters, such as septal thickness, LV end-diastolic diameter, LVEF, etc.

**Table 2:** CMR data in HOCM patients.

	Overall patients (n = 78)	Low sRGAE (n = 39)	High sRGAE (n = 39)	p value
LAD (mm)	39.7 ± 7.6	39.3 ± 5.8	40.2 ± 9.3	0.654
LVEDD (mm)	44.3 ± 3.8	44.7 ± 3.5	44 ± 4.1	0.45
Septal thickness (mm)	25(22-27)	24.5(21.3-26)	25(22-28)	0.575
LVEDV index (g/m^2^)	84.9(73.85-96.35)	81.1(72.8-96.2)	88.2(76.1-97.1)	0.225
LVESV index (g/m^2^)	29(25.5-33.9)	28.9(25.8-32.9)	29.1(25.3-35.8)	0.838
LVEF (%)	64.6 ± 6.4	64 ± 5.7	65.3 ± 7.0	0.4
LV mass index (g/m^2^)	92.4(69-106)	90.7(71.8-105.2)	92.4(66-116.3)	0.89
LGE,%	15.5 ± 8.5	16.6 ± 8.5	14.4 ± 8.4	0.264
**Feature Tracking**				
GRS (%)	24.6 ± 7.7	23 ± 7.7	26.3 ± 7.5	0.076
GCS (%)	14.7 ± 3.2	13.8 ± 3	15.5 ± 3.2	0.025
GLS (%)	9.9 ± 2.9	10 ± 2.7	9.8 ± 3.1	0.726
SRS (%)	17.8 ± 5.7	17.1 ± 5.7	18.5 ± 5.7	0.33
SCS (%)	12 ± 3.2	11.8 ± 2.9	12 ± 3.5	0.681
SLS (%)	4.6 ± 5.4	4.9 ± 5.4	4.3 ± 5.4	0.623

**Fig 5:**
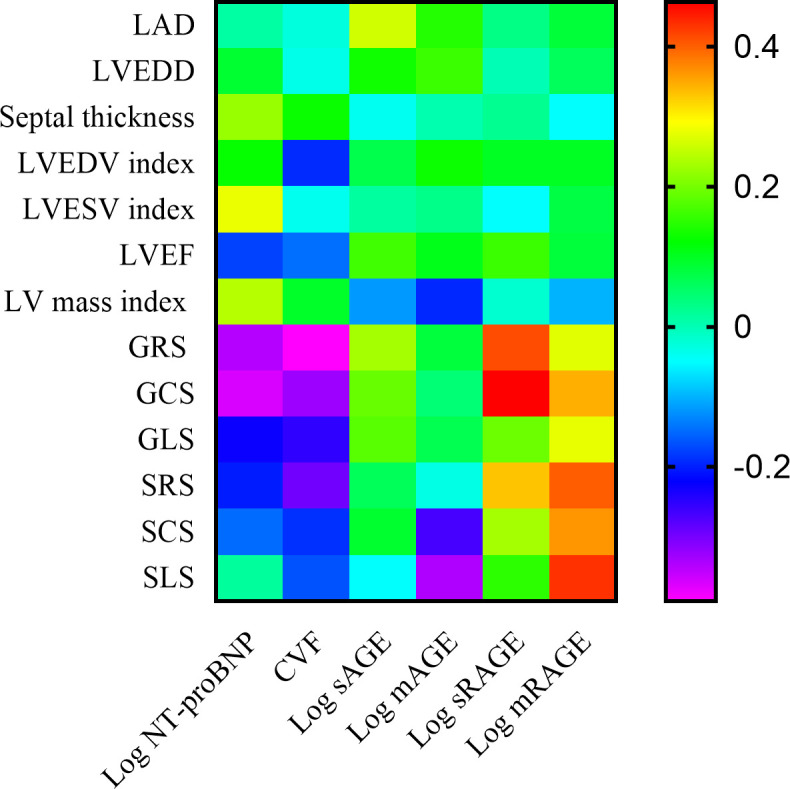
Correlations between LV function and biochemical variables.

GRS, global radial strains; GCS, global circumferential strains;GLS, global longitudinal strains; HOCM, hypertrophic obstructive cardiomyopathy; LAD, left atrial diameter; LV, left ventricular; EDD, end-diastolic diameter; EDV, end-diastolic volume; ESV, end-systolic volume; EF, ejection fraction; SRS, septal radial strains; SCS, septal circumferential strains;SLS, septal longitudinal strains.

log sRAGE was found to be significantly associated with GRS (r = 0.412, p < 0.01; Fig 4d), GCS (r = 0.463, p < 0.01; Fig 4e), and SRS (r = 0.331, p = 0.005; Fig 4f). As showed in Table 3, log mRAGE showed correlations with GCS (r = 0.345, p = 0.034), SRS (r = 0.4, p = 0.013), SCS (r = 0.362, p = 0.03), and SLS (r = 0.430, p = 0.009). Log sAGEs was also found to be correlated with left atrial diameters (r = 0.26, p = 0.031), while log mAGEs was associated with SLS (r = −0.339, p = 0.043). Furthermore, log NT-proBNP showed correlations with LVESV index (r = 0.278, p = 0.021), LV mass index (r = 0.242, p = 0.045), GRS (r = −0.343, p = 0.004), and GCS (r = −0.366, p = 0.002). Lastly, CVF was associated with GRS (r = −0.392, p = 0.001), GCS (r = −0.327, p = 0.006), GLS (r = −0.253, p = 0.036), and SRS (r = −0.297, p = 0.013). Fig 5 was presented as a heatmap to visually summarize these correlations.

**Table 3:** Correlations between myocardial function and biochemical variables.

	Log NT-proBNP	CVF	Log sAGE	Log mAGE	Log sRAGE	Log mRAGE
LAD	0.012	−0.027	0.260*	0.144	0.032	0.083
LVEDD	0.088	−0.035	0.131	0.159	0	0.06
Septal thickness	0.222	0.127	−0.041	0.004	0.024	−0.049
LVEDV index	0.125	−0.193	0.07	0.128	0.098	0.098
LVESV index	0.278*	−0.039	0.015	0.029	−0.049	0.075
LVEF	−0.177	−0.147	0.163	0.104	0.159	0.081
LV mass index	0.242*	0.093	−0.119	−0.195	−0.019	−0.101
GRS	−0.343*	−0.392*	0.232	0.08	0.412*	0.272
GCS	−0.366*	−0.327*	0.19	0.042	0.463*	0.345*
GLS	−0.228	−0.253*	0.179	0.067	0.193	0.276
SRS	−0.203	−0.297*	0.061	−0.033	0.331*	0.400*
SCS	−0.149	−0.189	0.089	−0.268	0.231	0.362*
SLS	0.016	−0.167	−0.049	−0.339*	0.15	0.430*

sAGEs/mAGEs, soluble/myocardial advanced glycation end products; CVF, collagen volume fraction; GRS, global radial strains; GCS, global circumferential strains;GLS, global longitudinal strains; HOCM, hypertrophic obstructive cardiomyopathy; LAD, left atrial diameter; LV, left ventricular; EDD, end-diastolic diameter; EDV, end-diastolic volume; ESV, end-systolic volume; EF, ejection fraction; NT-proBNP, N-terminal pro-brain natriuretic peptide; SRS, septal radial strains; SCS, septal circumferential strains;SLS, septal longitudinal strains; sRAGE/mRAGE, soluble/myocardial receptor for AGEs;

*Statistical significance

### 3.3. Prognostic value of log sRAGE

**Fig 6:**
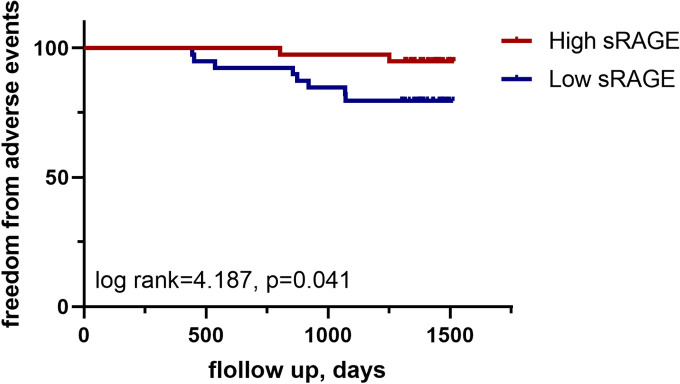
Survival of HOCM patients after septal myectomy according to the median of sRAGE.

During the follow-up of 3.8(3.6−4) years, 10 patients reached a cardiovascular endpoint, including 6 who admitted to the hospital because of new onset acute heart failure, 2 who had sustained new-onset atrial fibrillation and 2 who had acute coronary syndrome. Kaplan–Meier analysis showed significantly worse composite endpoint in high sRAGE than in low sRAGE(log rank = 4.187, p = 0.041; Fig 6). Univariate analyses showed that predictors of cardiovascular events in HOCM patients were diabetes (HR, 4.683; 95% CI, 1.209–18.143; p = 0.025), log NT-proBNP(HR, 43.067; 95% CI, 4.887–379.535; p = 0.001) and log sRAGE (HR, 0.005; 95% CI, 0–0.123; p = 0.001). Whereas, log NT-proBNP (HR, 35.975; 95% CI, 3.144–411.681; p = 0.004) and log sRAGE (HR, 0.013; 95% CI, 0.001–0.313; p = 0.007; Table 4) remained associated with the cardiovascular endpoint in multivariate analyses.

**Table 4:** Univariate and multivariable Cox regression analysis of HOCM patients for the prediction of cardiovascular endpoint after septal myectomy.

	Univariate analysis		Multivariate analysis	
	HR (95% CI)	P value	HR (95% CI)	P value
Age	0.997(0.950-1.046)	0.909	1.014(0.963-1.068)	0.593
Sex	1.744(0.492-6.180)	0.389	0.972(0.255-3.703)	0.972
Diabetes	4.683(1.209-18.143)	0.025	–	
Log NT-proBNP	43.067(4.887-379.535)	0.001	35.975(3.144-411.681)	0.004
Log sRAGE	0.005(0-0.123)	0.001	0.013(0.001-0.313)	0.007

NT-proBNP, N-terminal pro-brain natriuretic peptide; sRAGE, soluble receptor for advanced glycation end products;

## 4. Discussion

The present study revealed three novel findings: 1) Compared to healthy controls, HOCM patients had higher levels of sRAGE and sAGEs. Additionally, sRAGE was strongly correlated with mRAGE, while sAGEs did not correlate with mAGEs. 2) Both sRAGE and mRAGE were correlated with LV function and myocardial fibrosis. 3) sRAGE was identified as an independent predictor of adverse cardiac events in HOCM patients after septal myectomy.

### 4.1. AGEs/RAGE in circulation and myocardium

Usually, the concentration of AGEs accumulates with age, but its expression is markedly augmented in pathologic states such as diabetes and renal failure [17]. In our study, circulating AGEs were found to be higher in HOCM patients than in controls. Similar findings were observed in patients with heart failure, who showed a higher level of AGEs [18]. The circulating AGEs did not correlate with AGEs in myocardium, but AGEs in myocardium were found to be associated with RAGE in myocardium. This finding may indicate that circulating AGEs are derived from a wide variety of body tissues, and that the heart is not the primary source. In the myocardium, AGEs/RAGE reflected regional inflammation, which was more linearly related.

In our study, circulating RAGE was found to be increased in HOCM patients compared to controls. Furthermore, circulating RAGE was highly associated with RAGE in myocardium. RAGE is expressed at high levels in the lungs and skin of healthy adults, while low levels are expressed in the cardiovascular system [19,20].There are two RAGE isoforms, known jointly as sRAGE. One form of sRAGE, known as cRAGE, is generated at the cell surface by the proteolytic cleavage of RAGE at the boundary between its extracellular and transmembrane portions [21]. The other form, called esRAGE (for endogenous secretory RAGE), results from alternative splicing of RAGE pre-mRNA and accounts for less than 25% of total circulating sRAGE [22]. This RAGE ectodomain shedding is stimulated by inflammatory signals and cleaved by MMP9 [23]. This high correlation of RAGE in circulation and myocardium in our study suggests that inflammatory activation in the heart causes more transmembrane RAGE to be cleaved and released into circulation. However, due to the limited number of studies, it is difficult to draw a definite conclusion.

### 4.2. Correlation between AGEs/RAGE and LV function

Contradicting conventional assessment, strain analysis has emerged as a robust biomarker for assessing LV contractile performance, as it provides a direct measure of myocardial tissue deformation in HCM [24] Our study found that sRAGE was associated with global strain, while mRAGE was associated with septal strain. Furthermore, both sRAGE and mRAGE were observed to be inversely correlated with myocardial fibrosis. Despite earlier studies suggesting RAGE’s involvement in reactive oxygen species and inflammatory responses [25], our study indicates that RAGE is associated with LV remodeling by exerting an anti-fibrotic function. Scavello et al. [17]investigated the expression of RAGE mRNA and protein in young, middle-aged, and old mice. The authors discovered that RAGE expression was substantially low and remained unaltered with aging. Nonetheless, circulating RAGE was observed to decrease with aging. The administration of recombinant soluble receptor for advanced glycation end-products (sRAGE) was reported to reduce pro-fibrotic activities by inhibiting human cardiac fibroblast activity and their differentiation into myofibroblasts. Taken together, low levels of RAGE expression in myocardium suggest that RAGE signaling does not play a significant part in causing myocardial fibrosis. However, overexpression of RAGE in myocardium can lead to elevated levels of circulating RAGE in pathological situations, which could have an anti-fibrotic effect and promote improved LV function.

AGEs elicit their cellular effects through two primary mechanisms: extracellular and intracellular protein cross-linking, and cell surface receptor-mediated signaling. Our study found that, apart from a correlation between mAGEs and septal longitudinal strain, AGEs in circulation showed no significant correlation with LV function. Previously, we reported that mAGEs were involved in extracellular remodeling and protein cross-linking, increasing stiffness, and impairing LV function in HOCM [16]. Furthermore, AGEs can interact with several AGE receptors, such as RAGE, oligosaccharyltransferase-48, 80k-H phosphoprotein, and galectin-3 [4], which are broadly expressed in the body. Although AGE receptors mediate signaling leading to inflammation, we observed no correlation between soluble AGEs (sAGEs) and myocardial fibrosis or LV function in our study, implying a minimal effect of sAGEs on the heart.

### 4.3. The prognostic value of AGEs/RAGE

In our study, sRAGE was found to be an independent predictor for adverse events in HOCM patients after septal myectomy. Lower levels of RAGE were associated with a higher incidence of adverse events. However, conclusions from previous studies have not always been consistent. Studies have shown that high levels of sRAGE in individuals with pre-existing heart failure [26,27], coronary artery disease, and frailty were associated with a higher incidence of adverse cardiovascular events and/or mortality. Conversely, a negative association between total sRAGE or esRAGE and mortality has been reported in cancer [28]. In this respect, the clinical relevance of sRAGE has yet to be firmly established. Further research will be required to validate its value in larger and longitudinal cohorts.

### 4.4. Limitations

The soluble form of RAGE can be generated through alternative splicing (esRAGE), or from the cleavage of cell surface RAGE. Unfortunately, the ELISA assay currently used cannot distinguish between these two forms, limiting the ability to assess their respective pathophysiological functions. It is important to note that our results regarding AGE/RAGE are based solely on cross-sectional analysis, and therefore we cannot infer a causal relationship. Additionally, due to the relatively small number of participants in our study, we may not have had sufficient patients who experienced adverse events to adequately power a prognostic analysis. Third, genetic variants (e.g., AGER polymorphisms) or interactions with atrial fibrillation-related genes (e.g., PITX2) were not explored. Future studies should integrate genomic data to refine prognostic models. Furthermore, because myocardium samples were used, it was difficult to obtain levels of AGE/RAGE in healthy individuals. This limitation hindered our ability to accurately assess changes in the expression levels of AGE/RAGE in pathological situations.

## 5. Conclusions

This study indicates a potential role of AGE/RAGE in heart remodeling among HOCM patients. Specifically, circulating RAGE appears to act as a protective biomarker, as it is associated with better prognosis after septal myectomy, reducing fibrosis and improving heart function. It is plausible that higher circulating RAGE levels may be derived from higher expression levels in the myocardium.

## Supporting information

S1 File Measurement of biochemical biomarkers, Quantification of myocardial fibrosis, Cardiac surgery, CMR protocol and Analysis. (DOCX)

S2 File Data. (XLSX)
