# Supplementary material for: The role of advanced glycation end products (AGEs) and the receptor for AGEs (RAGE) in hypertrophic obstructive cardiomyopathy
Source: PLoS One. 2025 Jul 24;20(7):e0328032. doi: 10.1371/journal.pone.0328032 (PMC12289031; doi:10.1371/journal.pone.0328032)
Supplement: S1 File — (DOCX) [file pone.0328032.s001.docx]

**Supplemental material**

**Method**

**Measurement of biochemical biomarkers**

Venous blood samples were collected and centrifuged for serum at enrollment. Then, serum was taken and kept frozen at -70° C. Serum AGE(STA-817, Cell Biolabs Inc, San Diego, USA) and RAGE (DRG00, R&D Systems, Minneapolis, MN, USA) were determined by a commercial enzyme-linked immunosorbent assay (ELISA). NT-proBNP levels were measured by a commercial, fully automated, two-side electrochemiluminescence immunoassay (CobasE170, Roche, Basel, Switzerland).

The myocardium were directly collected after surgical myectomy and then were snap-frozen in liquid nitrogen and kept at -70°C for further analysis. Heart samples were homogenized in 1 mL of lysis buffer(Bestbio, Catalog No: BB-3209-2, China). The homogenate was centrifuged at 12000g for 15 minutes at 4°C, and the supernatant was collected. The supernatant was tested for AGE(STA-817, Cell Biolabs Inc, San Diego, USA) and RAGE (DRG00, R&D Systems, Minneapolis, MN, USA) with ELISA. Values of AGE and RAGE were standardized per 1 mg of heart tissue protein.

**Quantification of myocardial fibrosis**

Following dissection, the myocardium was fixed immediately in 10% formalin after being separated. The samples were cut into 5 µm thick sections after being embedded in paraffin. Masson’s trichrome staining was performed and the slides were enlarged 200× with light microscopy. Five fields of each slide were randomly selected to calculate myocardial fibrosis using Image-Pro Plus 6. 0 image analysis software (Media Cybernetics Inc, Buckinghamshire, UK). The collagen volume fraction (CVF) was calculated as the ratio of collagen-specific staining to the total area of the myocardium in each specimen.

**Cardiac surgery**

We applied extended septal myectomy evolving from the classic Morrow procedure. The hypertrophic ventricular septal leading to systolic anterior motion of the anterior mitral valve and left ventricular outflow tract(LVOT) gradient obstruction was resected. The resection range in the long-axis direction started from approximately 4 mm below the aortic ring to the apex of the left ventricle beyond the bases of the papillary muscles. In the shortaxis direction, the myectomy started rightward to the nadir of the right aortic cusp and to the left and terminated near the mitral anterior commissure. Part of the LV anterior free wall detached to the ventricular septal causing LVOT narrowing may also need to be resected. Furthermore, the anomalous chordal attachments between the mitral valve leaflets or papillary muscle and the ventricular septal were also excised. Additional surgery was performed based on expert consensus among the experienced cardiac surgeons. If intraoperative transoesophageal echocardiography detected a postoperative LVOT gradient >30 mmHg or more-than-moderate mitral valve regurgitation after weaning from cardiopulmonary bypass, reoperation was required

**CMR protocol and Analysis**

During the imaging procedure, four MRI-compatible electrodes were affixed between the right first and second ribs and the left fifth and sixth ribs of the subjects. Electrocardiogram (ECG) signals were acquired to synchronize the scanning at a specific time point. The epicardial and endocardial boundaries of the left ventricle (LV) and right ventricle (RV) myocardium were meticulously delineated throughout the complete cardiac cycle on every cine short-axis image. This facilitated the assessment of LV and RV end-diastolic and end-systolic volumes (EDV and ESV), end-diastolic diameter (EDD), ejection fractions (EF), and myocardial mass. Stroke volume (SV) represented the discrepancy between EDV and ESV. Myocardial mass was computed by multiplying the volume of the myocardium estimated at end-diastole by the specific gravity of the myocardium (1.05 g/ml). The indices for end-diastolic volume, end-systolic volume, and mass were indexed to the individual's body surface area. Subsequent to intravenous administration of 0.2 mmol/kg gadolinium-DTPA (Magnevist, Schering, Berlin, Germany), late gadolinium enhancement (LGE) images were acquired utilizing a phase-sensitive inversion recovery-spoiled gradient echo sequence, with image acquisition commencing 15 minutes post-administration. LGE images were captured in a standard short-axis view encompassing the entirety of the ventricle, as well as in long-axis views. The assessment of HCM morphology was performed by two experienced cardiologists, who independently reviewed the CMR images without knowledge of other clinical data.

Feature-tracking analysis was performed using the QStrain package (Medis Medical Imaging Systems), which offers precise ventricular anatomical tracking capabilities. The global longitudinal strains(GLS) were obtained by tracking the long horizontal axis cines whereas the circumferential(GCS) and radial strains(GRS) were derived from the short-axis cines on the standard CMR steady-state free precession sequence. The septal radial(SRS), circumferential(SCS), and longitudinal strains(SLS) were defined as the mean of peak strain from the basal-anteroseptal and mid-anteroseptal segments.
